# Prognostic value of ZEB-1 in solid tumors: a meta-analysis

**DOI:** 10.1186/s12885-019-5830-y

**Published:** 2019-06-27

**Authors:** Borong Chen, Baisheng Chen, Zhipeng Zhu, Weipeng Ye, Junjie Zeng, Gang Liu, Shengjie Wang, Jin Gao, Guoxing Xu, Zhengjie Huang

**Affiliations:** 1 Department of Gastrointestinal Surgery, Xiamen Cancer Hospital of The First Affiliated Hospital of Xiamen University, 55 Zhen Hai Road, Si Ming District, Xiamen, 361003 Fujian Province China; 2 Department of Endoscopy Center, Xiamen Branch of Affiliated Zhongshan Hospital of Fudan University, Xiamen, Fujian China; 3 Department of Gastrointestinal Surgery, First Clinical Medical College of Fujian Medical University, Fuzhou, Fujian China; 4 Department of Breast Surgery, The Third Hospital of Nanchang City, Key Laboratory of Breast Diseases, Nanchang, Jiangxi China; 5 Department of Endoscopy Center, First Affiliated Hospital of Xiamen University, Xiamen, China

**Keywords:** ZEB-1, Overall survival, Solid tumor, Meta-analysis

## Abstract

**Background:**

Zinc-finger E-box binding homeobox 1 (ZEB-1) plays crucial roles in epithelial-to-mesenchymal transition during tumor carcinogenesis. Published studies have examined the potential value of ZEB-1 as a biomarker for the prognosis of cancer. Nevertheless, the prognostic significance of ZEB-1 in human solid tumor remains inconclusive. Therefore, we performed the present meta-analysis to evaluate the prognostic value of ZEB-1 in patients with solid tumors.

**Methods:**

The 13 included studies (1616 patients) were exact electronic searched from Web of Science, PubMed and EBSCO until September 2018. Pooled hazard ratios (HR) and the corresponding 95% confidence intervals (CI) for overall survival (OS) were analyzed through random or fixed effects models. Univariate and multivariate analyses were independently performed. Subgroup analyses, heterogeneity and publication bias were investigated to further enhance reliability.

**Results:**

This research indicated that elevated expression of ZEB-1 significantly predicted worse OS in patients with solid tumors. In the univariate analysis, the pooled HR for OS was 1.66 (95% CI: 1.45–1.90; *P* < 0.01). Meanwhile, in multivariate analysis, the pooled HR for OS was 2.28 (95% CI: 1.58–3.30; *P* < 0.01). Begg’s funnel plot and Begg’s test did not show evidence of significant publication bias, both in univariate analysis and multivariate analysis.

**Conclusions:**

High expression of ZEB-1 was associated with poorer OS, suggesting that ZEB-1 may be a potential biomarker for the prediction of prognosis, and a novel therapeutic target in human solid tumors.

**Electronic supplementary material:**

The online version of this article (10.1186/s12885-019-5830-y) contains supplementary material, which is available to authorized users.

## Background

Zinc finger E-box binding homeobox 1 (ZEB-1) is a transcription factor, belongs to the human ZEB family. It is also known as dEF1, ZFHX1A, Nil-2-a, TCF8, AREB6, or BZP [1]. Functionally, ZEB-1 is implicated in multiple processes during the development of neural crest cells, lymphopoiesis, and neurogenesis. [2] Recently, further studies revealed that ZEB-1 plays a significant role in epithelial to mesenchymal transitions (EMT) during tumor invasion and metastasis in various types of human cancer. [3] EMT is a biological process characterized by the conversion from an epithelial cell phenotype to a mesenchymal phenotype, which is closely related to enhanced cell motility and invasion. [4] Research demonstrated that ZEB-1 promotes the process of EMT through regulation of the relevant protein binding domains, such as the p300-P/CAF binding domain, Smad interaction domain, and C-terminal-binding protein interaction domain. [2, 5] Furthermore, ZEB-1 facilitates EMT by suppressing the cell adhesion molecule E-cadherin, which is a critical transmembrane protein in maintaining the epithelial phenotype. [6, 7] This specific regulatory pathway is established as a hallmark of EMT. A study conducted by Hu et al. [8] indicated that ZEB-1through down-regulation of p21 transcription to promote the proliferation of breast cancer cells. Jia et al. [9] suggested that aberrant expression of ZEB-1in gastric cancer was associated with tumor stage, depth of invasion and poor differentiation. Accumulating evidence has demonstrated that ZEB-1was abnormally expressed in a variety of human solid tumors, and promoted an aggressive during carcinogenesis.

An increasing number of studies have shown that over-expression of ZEB-1 was related to shorter survival in several types of human solid tumors, including colorectal cancer, [10–12] gastric cancer, [13, 14] hepatocellular carcinoma, [15, 16] pancreatic cancer, [17, 18] esophageal squamous cell carcinoma, [19, 20] oral cavity carcinoma, [21] and intrahepatic cholangiocarcinoma. [22] Nevertheless, the prognostic significance of ZEB-1 in human solid tumor remains uncertain. Therefore, the aim of this comprehensive meta-analysis was to evaluate the prognostic role of ZEB-1expression for patients with solid tumor. This study was designed to assess the clinical value of ZEB-1 in terms of overall survival (OS) in patients with solid tumors, and shed more light on the development of ZEB-1 targeted therapy and prognostic indicators in this setting.

## Methods

### Literature search and selection criteria

An electronic literature retrieval of studies investigating the expression of ZEB-1 and clinical prognosis in Web of Science, PubMed and EBSCO was conducted until September 2018 to identify latent eligible articles. The search keywords for the subject heading terms were: “Zinc-finger E-box binding homeobox 1” or “ZEB-1” or “dEF1” or “ZFHX1A” or “Nil-2-a” or “TCF8” or “AREB6” or “BZP” and “cancer” or “tumor” or “neoplasm” or “carcinoma,” and “prognosis” or “survival”. Only studies involving patients with solid tumors were included. Collectively, 840 entries were identified.

The inclusion criteria were as follows: (1) diagnosis of human solid tumor; (2) the role of ZEB-1 in the tumor progression must be investigated; (3) description of the relevance of ZEB-1 expression to OS; (4) availability of data to obtain a hazard ratio (HR) and 95% confidence interval (CI); (5) publication in English. The exclusion criteria were as follows: (1) duplication; (2) conference abstract, review, and book; (3) basic research and animal experiments; (4) lack of usable data. There were two independent investigators (B.R Chen and Z.P Zhu) carefully scrutinized each candidate article. Disagreements were resolved through discussion between the two investigators until a consensus was reached.

### Data extraction and quality assessment

The following data were extracted by two independent investigators (B.S Chen and W.P Ye): name of first author, year of publication, country, type of cancer, case number, sex of patients, median age of patients, detection method, cut-off value of the overexpression of ZEB-1, follow-up period, survival analysis, obtained of HR, and Newcastle-Ottawa Scale (NOS) scores. Each HR and corresponding 95% CI were extracted based on the tables or Kaplan-Meier curves describing groups of patients with high or low expression of ZEB-1. Besides, all of the included researches were cohort studies. The NOS [23] was used to estimate the quality of the included studies in our meta-analysis. Studies with a NOS score ≥ 6 were defined as high quality research. Finally, any conflicting results were resolved through by discussion between the two authors.

### Statistical analysis

Survival outcomes were the most essential endpoints. Therefore, the HR and 95% CI were directly extracted from the original publications or calculated by means of the Kaplan– Meier method according to Tierney’s method. [24] Subsequently, the RevMan 5.3 analysis software (Cochrane Collaboration, Copenhagen, Denmark) was used to evaluate the prognostic value of ZEB-1 in solid tumors. The heterogeneity of pooled results was assessed through Higgin’s I^2^ statistics and Cochran’s *Q* test. [25, 26] I^2^ < 50% and *P* > 0.05 indicated the absence of severe heterogeneity and hence, a fixed-effects model (Mantel-Haenszel) was adopted. Otherwise, random-effects model (DerSimonian and Laird) was be performed (I^2^ ≥ 50% and *P* ≤ 0.05). Subgroup and sensitivity analyses were performed to evaluate the sources of heterogeneity. Begg’s funnel plot was conducted to estimate the risk of publication bias through STATA 12.0 software. A *P* < 0.05 denoted statistical significance.

## Results

### Study characteristics

**Fig. 1:**
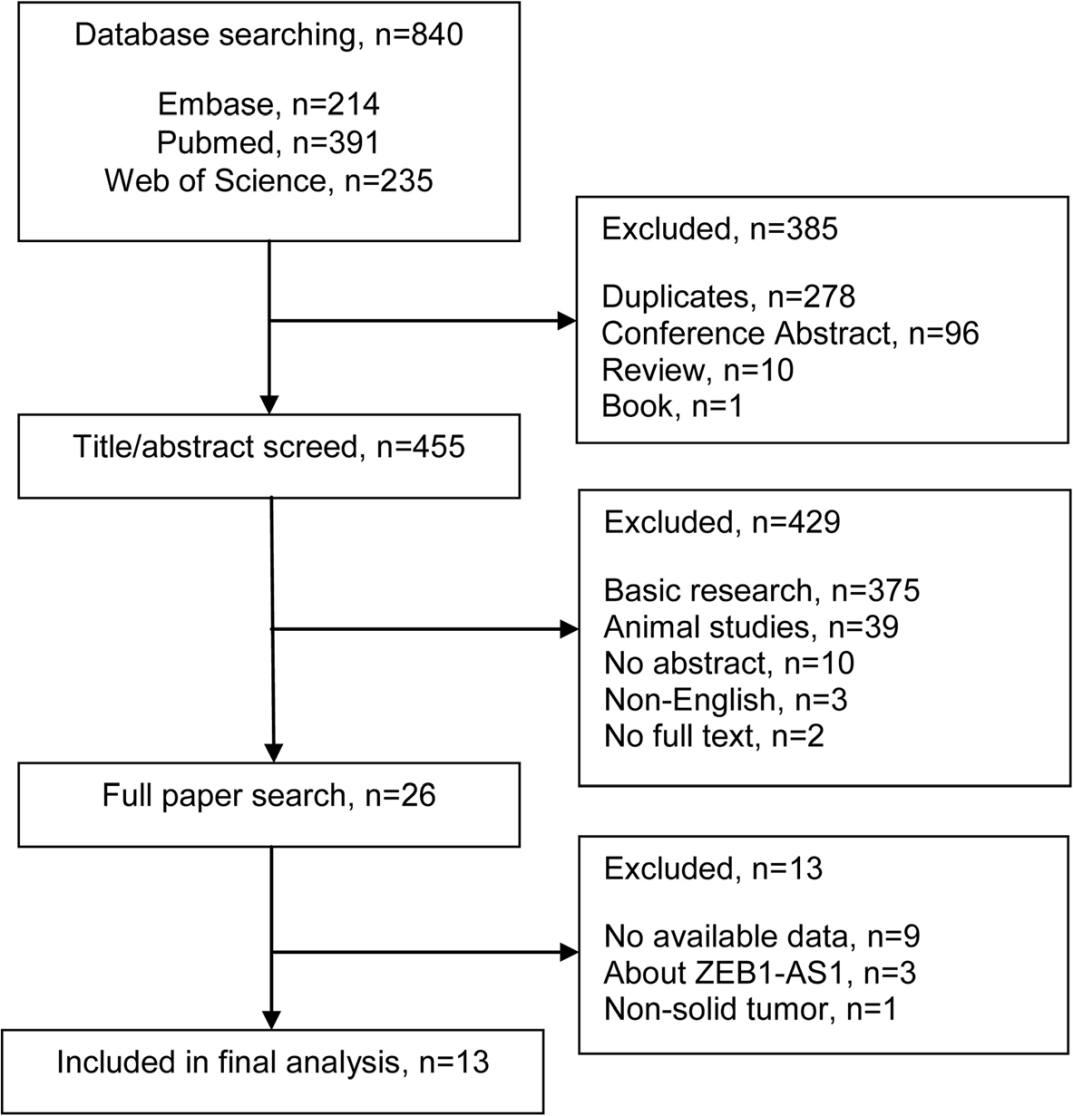
Flow diagram of the literature selection process

The detailed steps of literature selection are shown in Fig. 1. Initially, 840 relevant studies were identified. Eventually, 13 eligible articles published between 2011 and 2017 were included in this meta-analysis, and their main features are summarized in Table 1. A total of 1616 patients with solid tumors from China, Japan, United States of America, Germany, and Norway were analyzed. Besides, there were involved in seven types of solid tumors, including three colorectal cancer, [10–12] two gastric cancer, [13, 14] two hepatocellular carcinoma, [15, 16] two pancreatic cancer, [17, 18] two esophageal squamous cell carcinoma, [19, 20] one oral cavity carcinoma, [21] and one intrahepatic cholangiocarcinoma. [22] The included studies were performed to analyze OS. HR and 95% CI data were directly obtained from six studies. Data from remaining seven studies were extracted using Kaplan–Meier survival curves. All 13 studies reported a NOS score > 6 (Table 2).

**Table 1:** General characteristics of studies included in the meta-analysis

References	Country	Cancer type	Case No.	Male/Female	Age, median	Detect method	Cut-off	Follow-up months (Range)	Survival analysis	HR obtained	NOS score
Yao.X.F /2017^21^	China	OCC	120	75/45	57.6	IHC	score ≥ 7	66 (8–116)	OS (M)	Curve	8
Terashita.K /2016^22^	Japan	ICC	102	63/39	64	IHC	score > 40	35 (3–170)	OS (U)	Direct	8
Wu.D.W /2016^10^	China	CRC	145	NR	NR	IHC	score > 150	47.7 (3.4–85.7)	OS (M)	Direct	7
Goscinski.M /2015^19^	Norway	ESCC	151	92/59	NR	IHC	staining > 50%	60	OS (U)	Curve	7
Murai.T /2014^13^	Japan	GC	116	83/33	64	qRT-PCR	mRNA ≥0.0217	60	OS (M)	Curve	7
Yang.X.Z /2014^20^	China	ESCC	100	69/31	50	IHC	score > 4.90	32 (3–60)	OS (U)	Curve	8
Bronsert.P /2014^17^	Germany	PC	112	55/57	67	IHC	score ≥ 2	50	OS (U)	Direct	7
Hashiguchi.M /2013^15^	Japan	HCC	108	85/23	65.3	IHC	staining > 1%	60	OS (U) OS (M)	Direct Direct	8
Zhang.G.J /2013^11^	China	CRC	92	50/42	65	qRT-PCR	> median value	60	OS (U) OS (M)	Direct Direct	8
Okugawa.Y /2012^14^	Japan	GC	134	106/28	67	qRT-PCR	mRNA > 30.15	23 (1–79)	OS (U) OS (M)	Direct Direct	8
Zhou.Y.M /2012^16^	China	HCC	110	98/12	54	WB	expression> 30%	60	OS (M)	Curve	7
Kurahara.H /2012^18^	Japan	PC	76	52/24	67	IHC	staining≥10%	60	OS (U)	Curve	7
Singh.A /2011^12^	America	CRC	250	136/114	64.6	gene chip	>median value	45 (0.4–142)	OS (U)	Curve	6

Abbreviations: *ZEB-1* zinc finger E-box-binding homebox 1, *IHC* immunohistochemistry, *qRT-PCR* quantitative real time polymerase chain reaction, *WB* western blotting, *OS* overall survival, *M* multivariate analysis, *U* univariate analysis, *NR* not reported, *NOS* Newcastle-Ottawa scale, *OCC* oral cavity carcinoma, *ICC* intrahepatic cholangiocarcinoma, *CRC* colorectal cancer, *ESCC* esophageal squamous cell carcinoma, *GC* gastric cancer, *PC* pancreatic cancer, *HCC* hepatocellular carcinoma

**Table 2:** Quality assessment of eligible studies using the Newcastle-Ottawa Scale

References	Selection	Comparability	Outcome	NOS
Yao.X.F /2017^21^	★★★	★★	★★★	8
Terashita.K /2016^22^	★★★	★★	★★★	8
Wu.D.W /2016^10^	★★★	★	★★★	7
Goscinski.M /2015^19^	★★★	★★	★★	7
Murai.T /2014^13^	★★★	★	★★★	7
Yang.X.Z /2014^20^	★★★	★★	★★★	8
Bronsert.P /2014^17^	★★★	★★	★★	7
Hashiguchi.M /2013^15^	★★★	★★	★★★	8
Zhang.G.J /2013^11^	★★★	★★	★★★	8
Okugawa.Y /2012^14^	★★★	★★	★★★	8
Zhou.Y.M /2012^16^	★★★	★	★★★	7
Kurahara.H /2012^18^	★★★	★★	★★	7
Singh.A /2011^12^	★★★	★	★★	6

Abbreviations: *NOS* Newcastle-Ottawa scale

### Overall survival

**Fig. 2:**
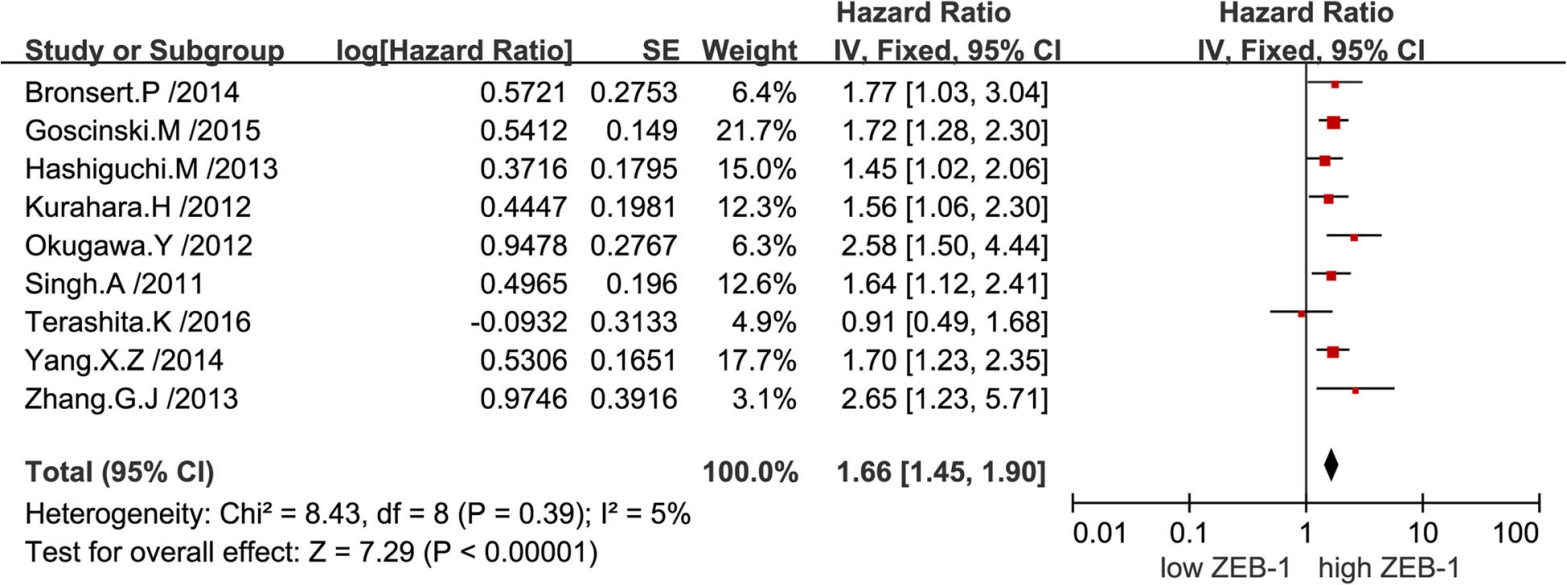
Forest plots showing the relationship between ZEB-1 and OS (univariate analysis)

**Fig. 3:**
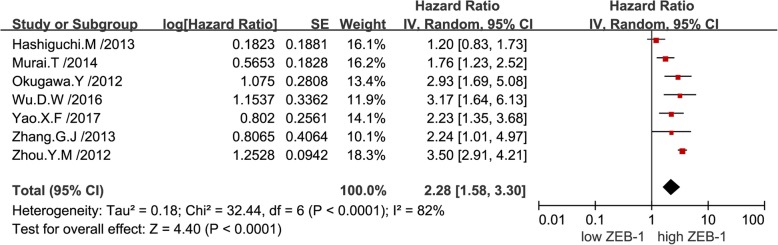
Forest plots showing the relationship between ZEB-1 and OS (multivariate analysis)

**Fig. 4:**
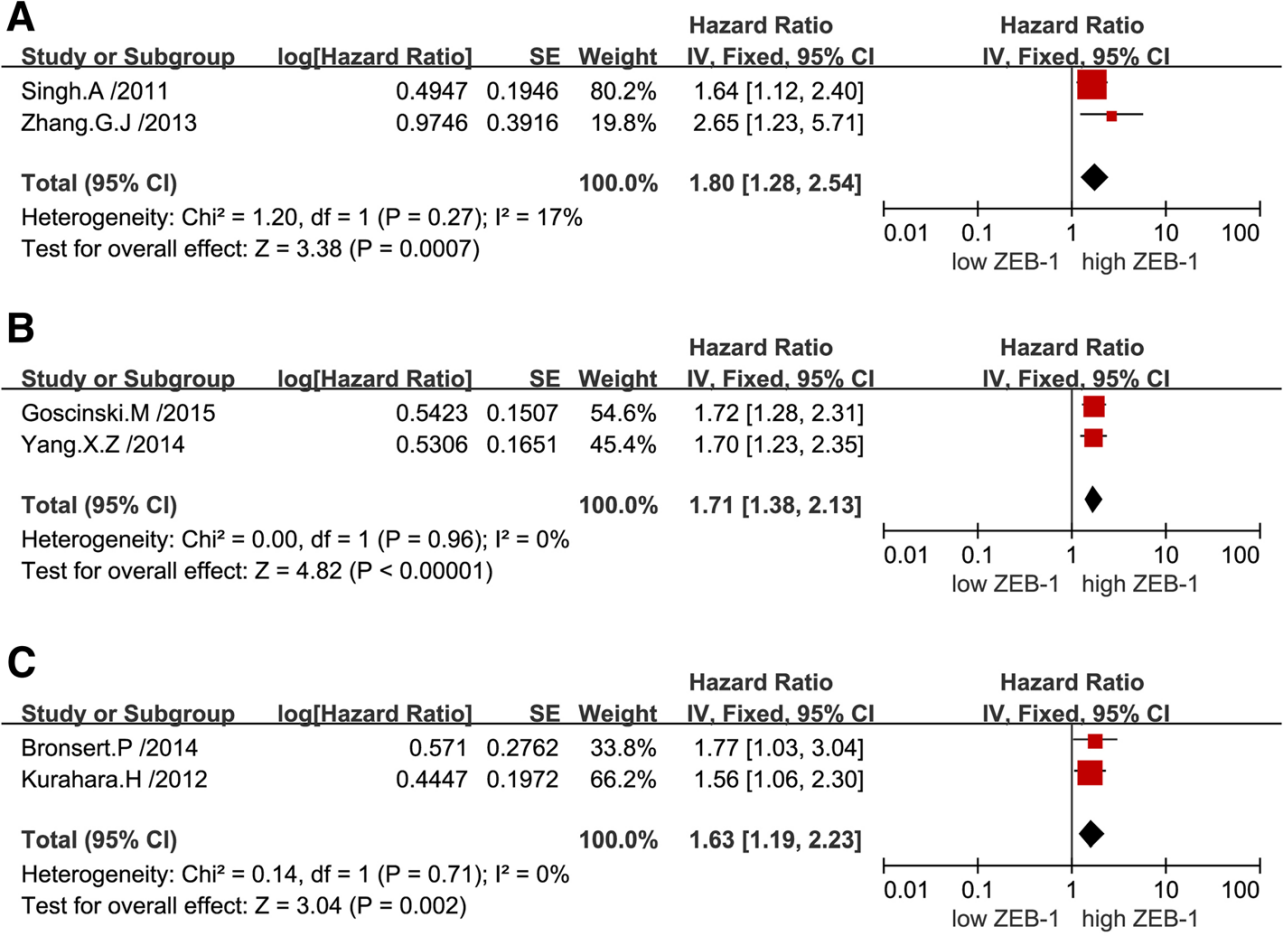
Subgroup analysis of OS (univariate analysis) according to the expression of ZEB-1 in various types of tumors. Colorectal cancer (**a**); Esophageal squamous cell carcinoma (**b**); Pancreatic cancer (**c**)

**Fig. 5:**
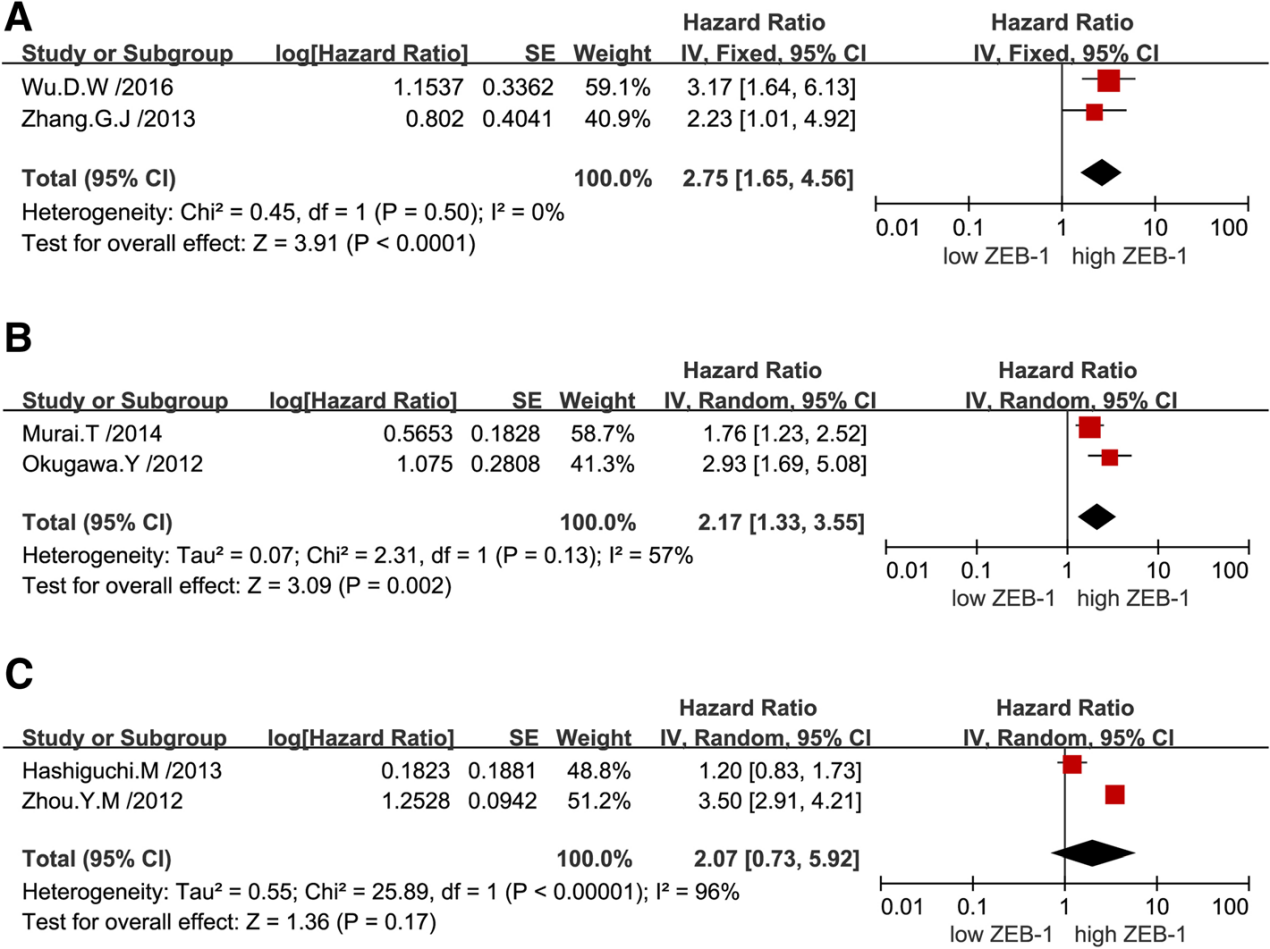
Subgroup analysis of OS (multivariate analysis) according to the expression of ZEB-1 in various types of tumors. Colorectal cancer (**a**); Gastric cancer (**b**); Hepatocellular carcinoma (**c**)

The combined analysis of published data from nine univariate analyses suggested that a high expression level of ZEB-1 was correlated with poor OS in patients with solid tumors (pooled HR: 1.66; 95% CI: 1.45–1.90). Notably, there was no significant heterogeneity reported (*P* = 0.39, I^2^ = 5%), thus, a fixed-effects model was performed (Fig. 2).The combined analysis of published data from seven multivariate analyses demonstrated that overexpression of ZEB-1 was associated with worse survival outcome in patients with solid tumors (pooled HR: 2.28; 95% CI: 1.58–3.30), and there was significant heterogeneity found (*P* < 0.01, I^2^ = 82%), hence, a random-effects model was utilized (Fig. 3). Subsequently, a sensitivity analysis was performed to explore the sources of heterogeneity. Exclusion of any individual study from the analysis did not significantly change the results (data not shown). Furthermore, a subgroup analysis was conducted based on various types of cancer to detect heterogeneity. The results indicated a negative effect of high ZEB-1 expression on OS (univariable analysis) in patients with colorectal cancer (pooled HR: 1.80; 95% CI: 1.28–2.54) (Fig. 4a), esophageal squamous cell carcinoma (pooled HR: 1.71; 95% CI: 1.38–2.13) (Fig. 4b) and pancreatic cancer (pooled HR: 1.63; 95% CI: 1.19–2.23) (Fig. 4c). In addition, a similar result was found on OS (multivariate analysis) in patients with colorectal cancer (pooled HR: 2.75; 95% CI: 1.65–4.56) (Fig. 5a), gastric cancer (pooled HR: 2.17; 95% CI: 1.33–3.55) (Fig. 5b) and hepatocellular carcinoma (pooled HR: 2.07; 95% CI: 0.73–5.92) (Fig. 5c). Additionally, subgroup analysis of OS according to different detection methods of ZEB-1 expression showed that high levels of ZEB-1 protein and mRNA expression were both correlated with poor OS in patients with solid tumors (Additional file 1).

### Publication bias

**Fig. 6:**
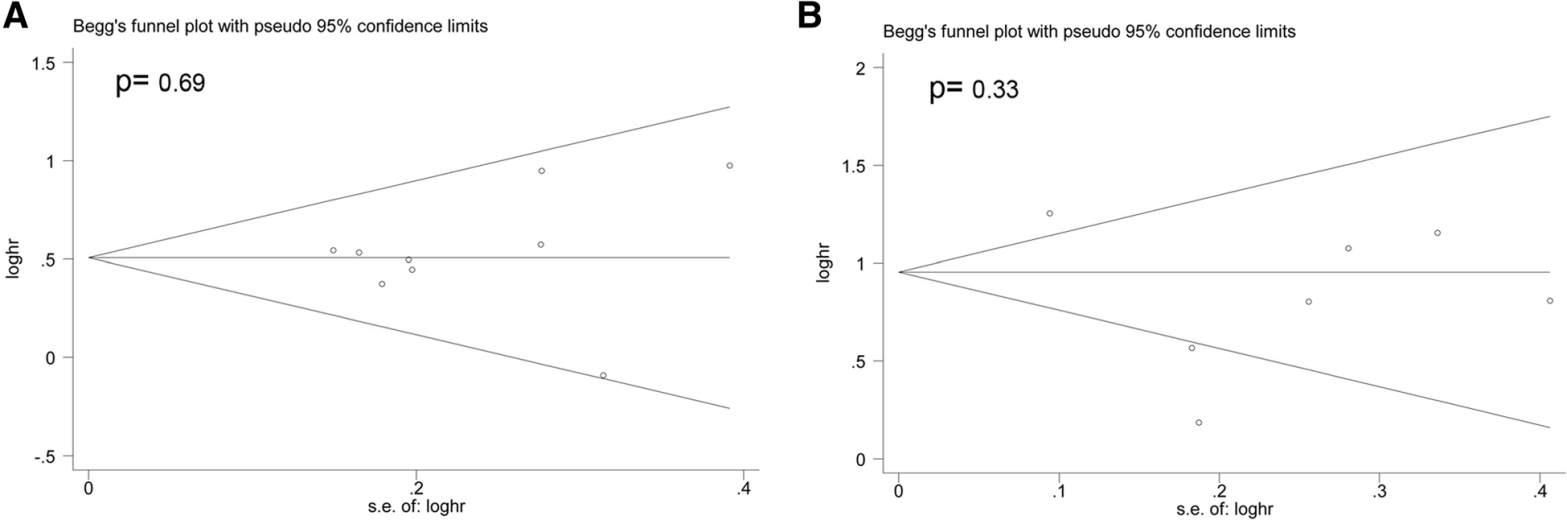
Funnel plot evaluating potential publication bias for OS in the univariate (**a**) and multivariate (**b**) analyses

In this study, Begg’s funnel plot and Begg’s test were performed to evaluate potential publication bias. The data indicated that there was no significant publication bias for OS in both the univariate (Fig. 6a) and multivariate analyses (Fig. 6b).

## Discussion

ZEB-1 is a 190-210kD protein encoded by the TCF8 gene, which is an important inducer of EMT and characterized as the repressor of cell adhesion molecules as well as cell polarity correlative genes. [3, 27] Numerous published studies indicated that EMT is an essential mechanism during tumor progression, invasion and metastasis. [28, 29] Unfortunately, metastases and invasions are highly characteristic of malignant tumors. Verschueren et al. [30] reported that ZEB-1 interacts with Smad proteins through mediation of transforming growth factor-β signaling to facilitate EMT. Singh et al. [31] revealed that high expression of ZEB-1 may have an impact on cell-cell interactions resulting in endometrial cancer cell invasion and metastasis. The growth inhibitory effects of ZEB-1may repress the expression of members of the miR-200 family, which function as strong inducers of epithelial differentiation. [32, 33] Knockdown of ZEB-1 may induce cell apoptosis, while high ZEB-1 expression may drastically suppress lung cancer cells, as shown through soft agar colony formation assays. [34] Krishnamachary et al. [35] demonstrated that the expression of ZEB-1was regulated through the hypoxia-inducible factor 1/ E-cadherin signaling pathway in renal clear cell carcinoma. Moreover, other studies depicted the non-EMT functions of ZEB-1, indicating its critical role in regulating cell cycle progression, apoptosis, and senescence. [36] These previous studies demonstrated that ZEB-1is involved in the malignant progression of various types of human cancer through complicated molecular mechanisms.

An increasing number of research studies reported that the level of ZEB-1 expression has a crucial impact on patient survival. Yang et al. [20] reported that down regulation of ZEB-1 significantly reduced the invasive and migratory abilities of esophageal squamous cell carcinoma. In vitro, high expression of ZEB-1 was associated with poor OS in patients with esophageal squamous cell carcinoma. In gastric cancer patients, increased expression of ZEB-1 was markedly correlated with peritoneal dissemination and worse clinical prognosis. [14] Yao et al. [21] identified that high level of ZEB-1 expression was associated with recurrence, lymph node metastasis, worse pathologic grading and low survival rates in oral cavity carcinoma. Therefore, ZEB-1may serve as an effective prognostic marker and a promising novel therapeutic target in patients with solid tumors. Accordingly, we conducted this meta-analysis to evaluate the prognostic value of ZEB-1.

In the present study, we systematically assessed the OS data of 1616 patients with solid tumors included in 13 eligible articles. The results provided strong evidence that overexpression of ZEB-1was significantly correlated with shorter OS in both univariate and multivariate analyses. Regarding the types of tumors, our study indicated that high expression of ZEB-1 was also significantly associated with worse OS in colorectal cancer, esophageal squamous cell carcinoma, pancreatic cancer, gastric cancer and hepatocellular carcinoma. This combined data analysis suggested that ZEB-1 may be a potential prognostic marker and therapeutic target for most solid tumors. Thus, it is essential to further investigate the clinical features and therapeutic implications of ZEB-1 expression in carcinomas.

This meta-analysis is characterized by several limitations. Firstly, only published studies and articles in English were included, and small sample studies with negative results may not have been published, which inevitably account for publication bias and selection bias. Secondly, the methods of measurement method and cut-off values for the evaluation of ZEB-1 expression were discordant. Finally, HR extracted from Kaplan–Meier curves appear to be no more reliable than those reported directly.

## Conclusions

In conclusion, overexpression of ZEB-1 is correlated with poor OS in most solid tumors. This finding suggests that ZEB-1 may be a potential prognostic biomarker, and a novel therapeutic target in human solid tumors.

## Additional file

Additional file 1: Subgroup analysis of overall survival according to different detection methods of ZEB-1. Protein (A) and mRNA (B) levels. (JPG 923 kb)

## Data Availability

The datasets used and analyzed in the present study are available from the corresponding author upon reasonable request.

## Abbreviations

CI: Confidence intervals
EMT: Epithelial to mesenchymal transitions
HR: Hazard ratios
OS: Overall survival
ZEB-1: Zinc-finger E-box binding homeobox 1
