# Supplementary figures and images for: Prognostic value of ZEB-1 in solid tumors: a meta-analysis
Source: BMC Cancer. 2019 Jun 27;19:635. doi: 10.1186/s12885-019-5830-y (PMC6598232; doi:10.1186/s12885-019-5830-y)

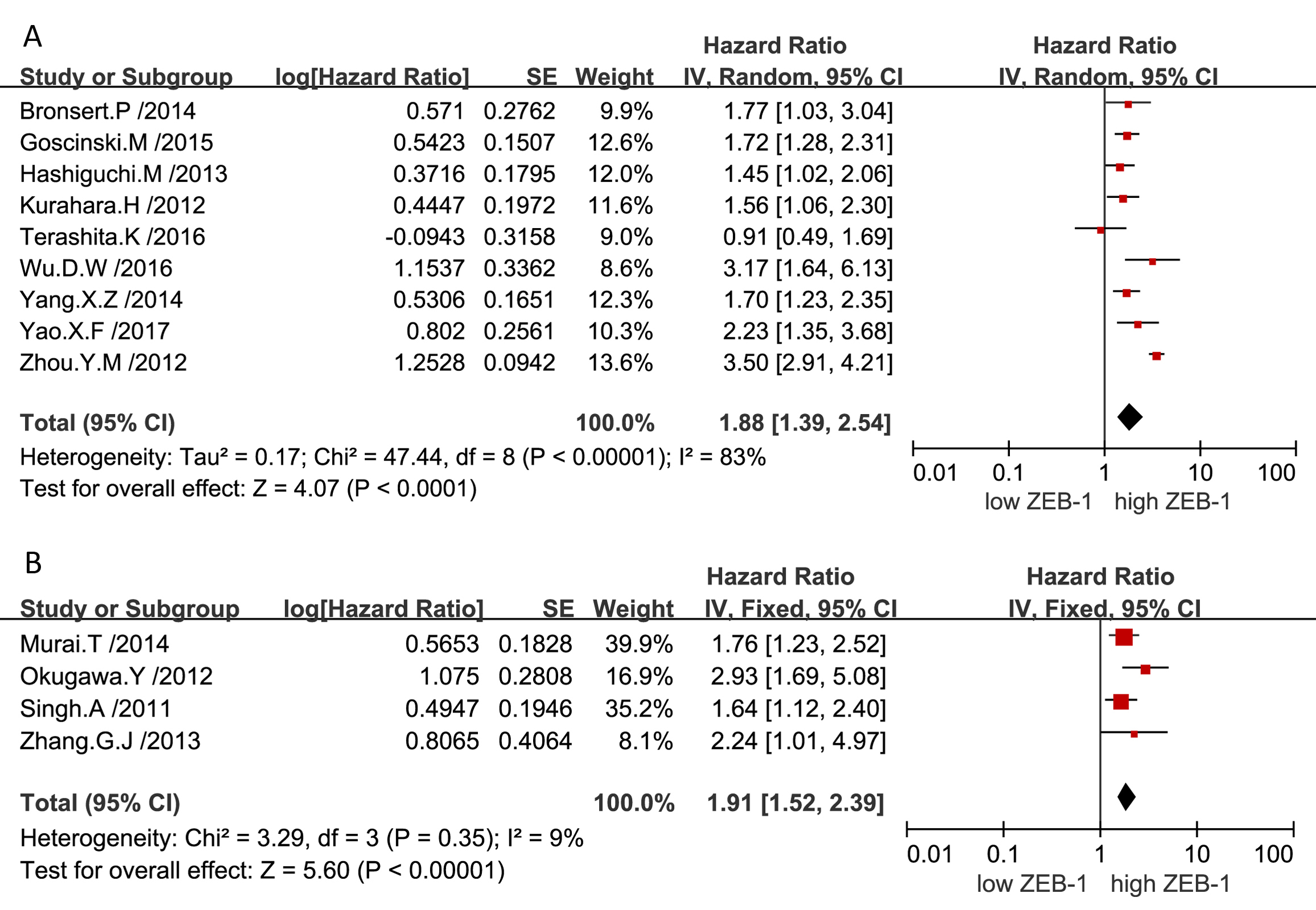

Supplement: Supplementary file 1 — Subgroup analysis of overall survival according to different detection methods of ZEB-1. Protein (A) and mRNA (B) levels. (JPG 923 kb) [file 12885_2019_5830_MOESM1_ESM.jpg]
